# Health‐related quality of life in patients with multiple system atrophy using the EQ‐5D‐5L

**DOI:** 10.1002/brb3.2774

**Published:** 2022-09-19

**Authors:** Yi Xiao, Lingyu Zhang, Qianqian Wei, Ruwei Ou, Yanbing Hou, Kuncheng Liu, Junyu Lin, Tianmi Yang, Huifang Shang

**Affiliations:** ^1^ Department of Neurology, Rare Disease Center, Laboratory of Neurodegenerative Disorders, National Clinical Research Center for Geriatric, West China Hospital Sichuan University Chengdu China

**Keywords:** depression, fatigue, health‐related quality of life, multiple system atrophy

## Abstract

**Background:**

Multiple system atrophy (MSA) is an incurable neurodegenerative disease. We aimed to investigate the health‐related quality of life (HRQoL) and the determinants of HRQoL in patients with MSA.

**Methods:**

The five‐level EuroQol five‐dimensional questionnaire (EQ‐5D‐5L) was used to evaluate patients’ HRQoL. The results of HRQoL were indicated by the EQ‐5D‐5L index values and visual analog scale (EQ VAS) scores. Specific scales were used to measure disease severity, cognition, frontal lobe function, anxiety, depression, fatigue, and sleep disorders. The beta mixture model and the linear regression model were used to explore the determinants of HRQoL in patients with MSA.

**Results:**

A total of 205 patients with cerebellar variants (MSA‐C; 53.9%) and 175 patients with parkinsonian variants (MSA‐P; 46.1%) were included in this cross‐sectional study. The mean values of the EQ‐5D‐5L index values and EQ VAS scores were .558 and 59.5, respectively. Problem with mobility was the problem reported by the highest proportion of patients (92.1%), followed by problems with usual activities (88.7%), self‐care (81.3%), anxiety/depression (72.1%), and pain/discomfort (53.9%). The determinants of the lower EQ‐5D‐5L index values in patients with MSA were greater disease severity, fatigue, Parkinson's disease‐related sleep problems (PD‐SP), depressive mood, and anxious mood. Greater disease severity, fatigue, and depressive mood were associated with lower EQ VAS scores.

**Conclusion:**

The problem reported most frequently by Chinese individuals with MSA was mobility. In addition to the greater disease severity of MSA, fatigue, PD‐SP, depression, and anxiety were determinants of poor HRQoL.

## 1 INTRODUCTION

Multiple system atrophy (MSA) is a rare, rapidly progressive neurodegenerative disease featuring autonomic failure, parkinsonism, and/or cerebellar ataxia. To date, the etiology of MSA is still uncertain, and no cure for MSA has been developed. MSA can be subclassified into a cerebellar variant (MSA‐C) and a parkinsonian variant (MSA‐P) according to the predominant motor symptoms (Gilman et al., 2008). In addition to experiencing severe disability in a short duration, many patients experience various nonmotor symptoms, including urinary incontinence, dysarthria, depression, anxiety, sleep disorders, and so on. In addition, nonmotor symptoms, such as depression, anxiety, and autonomic deficits, have a negative impact on patients’ lives and lead to a heavy disease burden (Benrud‐Larson, Sandroni, Schrag, & Low, 2005; Du et al., 2018; Schrag et al., 2006; Winter et al., 2011).

Patients with MSA were reported to have a poorer health‐related quality of life (HRQoL) than healthy controls and patients with Parkinson's disease (PD) (Jecmenica‐Lukic, Pekmezovic, Petrovic, Dragasevic, & Kostić, 2018; Schrag et al., 2006). The five‐level EuroQol five‐dimension questionnaire (EQ‐5D‐5L), a preference‐based instrument developed by the EuroQol Group, is one of the most frequently used tools to evaluate HRQoL worldwide (Herdman et al., 2011). The EQ‐5D‐5L has been utilized to evaluate HRQoL in various patient populations with chronic diseases (Seng et al., 2020; Wee et al., 2016). The EQ‐5D‐5L was developed from the three‐level version of the EQ‐5D (EQ‐5D‐3L) and was indicated to have some advantages over the EQ‐5D‐3L (Janssen, Bonsel, & Luo, 2018; Thompson & Turner, 2020). The EQ‐5D‐5L had increased sensitivity, higher precision, lower bias, and decreased ceiling effect in measuring health status compared to the EQ‐5D‐3L (Janssen et al., 2018; Thompson & Turner, 2020). In addition, EQ‐5D‐5L was proved to have good evidence of discriminant validity, while EQ‐5D‐3L was less sensitive in patients with PD (Xin & McIntosh, 2017). A few studies have focused on the HRQoL of MSA by using the EQ‐5D‐3L (Du et al., 2018; Higginson et al., 2012; Schrag et al., 2006; Winter et al., 2011). These studies found that the most frequently affected dimensions in MSA were mobility, self‐care, and usual activities (Du et al., 2018; Higginson et al., 2012; Schrag et al., 2006; Winter et al., 2011). In addition to the severity of the disease (Jecmenica‐Lukic et al., 2018; Schrag et al., 2006; Winter et al., 2011; Zhang et al., 2017), some studies found that depression was the determinant of the HRQoL of patients with MSA (Du et al., 2018; Schrag et al., 2006). However, no study evaluated the HRQoL of MSA patients with the EQ‐5D‐5L.

Therefore, in the current study, we aimed to fill the gap in evaluating the HRQoL of MSA using the EQ‐5D‐5L. Additionally, we used specific scales to measure the severity of nonmotor symptoms, including cognition, frontal lobe dysfunction, depression, anxiety, sleep disorders, and fatigue. We aimed to explore the determinants of HRQoL in patients with MSA and the differences between MSA‐C and MSA‐P.

## 2 METHOD

### 2.1 Patient

Consecutive patients were recruited from the Department of Neurology, West China Hospital, Sichuan University, from March 2018 to July 2021. All patients met the diagnostic criteria of probable MSA through a comprehensive medical history review and physical examination (Gilman et al., 2008). The exclusion criteria were as follows: (1) magnetic resonance imaging scan, spinal cerebellar ataxia genetic tests (SCA1, 2, 3, 6, 7), or blood test indicating a diagnosis of other neurological diseases; (2) could not complete the interview because of dysarthria, weakness, or other reasons. This study was approved by the Ethics Committee of West China Hospital of Sichuan University, and informed consent forms were signed by all patients.

### 2.2 Clinical symptoms evaluation

The clinical data were collected by experienced neurologists during a face‐to‐face interview. The demographic characteristics, including age, sex, education, disease duration, and age of onset, were collected. Disease severity was measured by the Unified Multiple System Atrophy Rating Scale (UMSARS) (Wenning et al., 2004). The UMSARS was composed of four parts and the total score was calculated by the sum of scores of UMSARS part Ⅰ (historical review) and UMSARS part Ⅱ (motor examination scale) with a higher score indicating more severe disease. The UMSARS part III (autonomic examination) recorded blood pressure values of orthostatic challenge and the existence of orthostatic symptoms. The UMSARS part IV evaluated the global disability of patients with a higher stage indicating a worse disability. Orthostatic hypotension was defined as a drop in systolic blood pressure ≥30 mm Hg and/or diastolic blood pressure ≥15 mm Hg. Global cognitive function was evaluated using the Montreal Cognitive Assessment (MoCA) (Nasreddine et al., 2005). Cognitive impairment was defined as MoCA scores <19 for individuals with no more than 6 years of education, MoCA scores <22 for individuals with 7–12 years of education, and MoCA scores <24 for individuals with more than 12 years of education (Chen et al., 2016). Frontal lobe dysfunction was defined as a Frontal Assessment Battery score <16 (Dubois, Slachevsky, Litvan, & Pillon, 2000). The depressive and anxiety emotions were, respectively, defined as Hamilton Depression Rating Scale (24 items) scores ≥8 (Hamilton, 1967) and Hamilton Anxiety Rating Scale scores ≥6 (Clark & Donovan, 1994). Fatigue was defined as a mean Fatigue Severity Scale score ≥4 (Friedman et al., 2010). Parkinson's disease‐related sleep problems were defined as a Parkinson's Disease Sleep Scale 2nd version score ≥18 (Horvath et al., 2014). Excessive daytime sleepiness was defined as an Epworth Sleepiness Scale score ≥10 (Johns, 1991). Rapid eye movement sleep behavioral disorder (RBD) was defined as a Rapid Eye Movement Sleep Behavior Disorder Screening Questionnaire score ≥5 (Stiasny‐Kolster et al., 2007).

### 2.3 Assessment of HRQoL

The HRQoL was evaluated using the Chinese version of EQ‐5D‐5L (Luo et al., 2013). The EQ‐5D‐5L was completed by patients during the face‐to‐face interviews. The EQ‐5D‐5L contains a questionnaire with five dimensions and a vertical visual analog scale (EQ VAS). The five dimensions are mobility, self‐care, usual activities, pain/discomfort, and anxiety/depression. Each dimension has five levels ranging from “no problems,” “slight–moderate–severe” problems, to “extreme problems/unable to.” Descriptive levels of each dimension were dichotomized to “no problems” (level one) and “problems” (level two to five). The five‐digit health state profile was converted to EQ‐5D‐5L index values according to the EQ‐5D‐5L value set for China (Luo et al., 2017). The EQ‐5D‐5L index values are anchored at 0 (death) and 1 (perfect health) and can be used to assess quality‐adjusted life‐years. The results of the EQ VAS were self‐rated scores marked from 0 to 100, reflecting the patient's perception of their own overall health on the day of the interview. The EQ‐5D‐5L index value set of the current study ranged from −.391 to 1.000, with 1.000 indicating a state of full health, 0 indicating death, and a negative value indicating that the health state was worse than death.

### 2.4 Statistical analysis

First, the Kolmogorov–Smirnov test was performed as the normality test. Since the data were not normally distributed, the Mann–Whitney test was conducted to compare the EQ‐5D‐5L index values and EQ VAS scores of patients in different subgroups regarding sex, subtype, frontal lobe dysfunction, cognitive impairment, depressive emotion, anxiety emotion, excessive daytime sleepiness, RBD, PD‐SP, and fatigue. Mann–Whitney test or Chi‐square test was conducted to compare demographic characteristics between MSA‐C and MSA‐P for continuous and categorical variables.

Spearman's correlation analysis was conducted to explore relationships between the EQ‐5D‐5L index values and scores on the clinical symptoms scale. Since the distribution of the EQ‐5D‐5L utility value was skewed and featuring with truncations (see Table S1), the beta mixture regression model was used to analyze the associated factors of EQ‐5D‐5L utility values. (Gray & Alava, 2018). All the demographic and clinical symptoms were included in the mixture regression models to explore the determinants of EQ‐5D‐5L utility values. Stepwise multivariate linear regression analysis was used to explore the potential determinants of EQ VAS scores in the total MSA group and two subtypes. Demographic variables with *p* < .1 in the univariate models (age, sex, education level, and disease duration) and all the clinical symptoms were included in the multivariate models. Clinical symptoms were included in the two kinds of regression models as dichotomous variables according to the cutoff score of scales as described before; *p* < .05 was considered statistically significant. SPSS 22.0 (SPSS, Inc., Chicago, IL, USA) and Stata 16.0 (StataCorp) were used to perform data analysis.

## 3 RESULTS

### 3.1 Clinical characteristics of MSA and two subtypes

Finally, 205 MSA‐C (53.9%) and 175 MSA‐P (46.1%) were included in the study. The clinical characteristics of the patients are displayed in Table 1. The average age and disease duration were 60.3 and 2.6 years, respectively. There were 212 male patients (55.8%) and 168 females (44.2%). Patients with MSA‐P had older age and age of onset and higher scores on the Parkinson's Disease Sleep Scale 2nd version and the Epworth Sleepiness Scale than patients with MSA‐C. Patients with MSA‐C had a higher proportion of orthostatic hypotension and higher Rapid Eye Movement Sleep Behavior Disorder Screening Questionnaire scores than patients with MSA‐P. There were no significant differences in other clinical features between the two subtypes.

**TABLE 1:** Demographic and clinical characteristics of patients with MSA and two subtypes

	Total (*n* = 380)	MSA‐C (*n* = 205)	MSA‐P (*n* = 175)	*p*‐value
Sex (male)	212 (55.8%)	117 (57.1%)	95 (54.3%)	.586
Age (years)	60.9 (53.6−66.7)	57 (52.7−64.5)	63.3 (55.1−68.6)	<.001*
Age of onset (years)	58.5 (51−64.1)	54.8 (50.1−61.8)	60.5 (52.6−65.7)	<.001*
Disease duration (years)	2.4 (1.5−3.4)	2.3 (1.5−3.2)	2.6 (1.5−3.5)	.395
Education (years)	9 (7−12)	9 (7−12)	9 (7−12)	.898
UMSARS‐Ⅰ	16 (12−21)	16 (12−21)	15 (11−21)	.458
UMSARS‐Ⅱ	18 (13−23)	18 (13−23)	18 (14−23)	.375
UMSARS‐IV	2 (1−3)	2 (1−3)	2 (1−3)	.988
UMSARS‐total	33 (26−43)	33 (26−42.5)	33 (25−43)	.921
FAB	15 (13−17)	15 (13−17)	15 (13−17)	.682
MoCA	23 (19−26)	23 (19−26)	23 (19−26)	.552
FSS	44 (18−54)	44 (16−54)	44 (21−54)	.273
PDSS‐2	10 (6−15)	9 (5−14)	12 (7−18)	<.001*
ESS	5 (2−8)	4 (2−7)	6 (3−10)	.005*
RBDSQ	5 (2−9)	6 (2.5−9)	4 (2−8)	.009*
HAMD	12 (6−18)	12 (5−18)	12 (6−18)	.827
HAMA	9 (5−15)	9 (5−14)	9 (5−16)	.558
OH	155 (40.8%)	94 (45.9%)	61 (34.9%)	.030*

*Note*: Continuous variables are displayed as median and quartile. Dichotomous variables are displayed as numbers and percentages. Mann–Whitney test or Chi‐square test was performed comparing characteristics between MSA‐C and MSA‐P.

Abbreviations: ESS, the Epworth Sleepiness Scale; FAB, the frontal assessment battery; FSS, the Fatigue Severity Scale; HAMA, the Hamilton Anxiety Rating Scale; HAMD, the Hamilton Depression Rating Scale; MoCA, the Montreal Cognitive Assessment; MSA, multiple system atrophy; MSA‐C, MSA with predominately cerebellar ataxia; MSA‐P, MSA with predominately parkinsonism; OH, orthostatic hypotension; PDSS‐2, the Parkinson's Disease Sleep Scale 2nd version; RBDSQ, Rapid Eye Movement Sleep Behavior Disorder Screening Questionnaire; UMSARS, the Unified Multiple System Atrophy Rating Scale.

^*^Significant difference.

### 3.2 EQ‐5D‐5L in MSA and different subgroups

**FIGURE 1:**
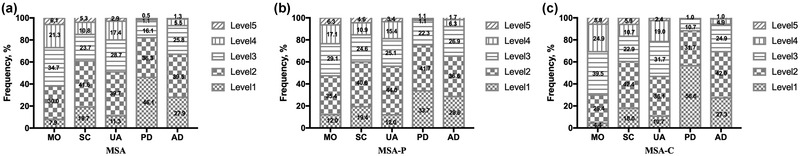
Distribution of five levels in each dimension of EQ‐5D‐5L of (a) total patients with MSA, (b) patients with MSA‐P, and (c) patients with MSA‐C. Level 1: no problems; level 2: mild problems; level 3: moderate problems; level 4: severe problems; level 5: extreme problems. EQ‐5D‐5L, the five‐level EuroQol five‐dimension questionnaire; MSA, multiple system atrophy; MSA‐P, MSA with predominately parkinsonism; MSA‐C, MSA with predominately cerebellar ataxia; MO, mobility; SC, self‐care; UA, usual activities; PD, pain/discomfort; AD, anxiety/depression

**FIGURE 2:**
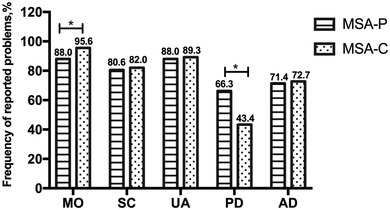
Frequency of reported problems for each dimension of EQ‐5D‐5L between MSA‐P and MSA‐C patients. *Significant difference. EQ‐5D‐5L, the five‐level EuroQol five‐dimension questionnaire; MSA, multiple system atrophy; MSA‐P, MSA with predominately parkinsonism; MSA‐C, MSA with predominately cerebellar ataxia; MO, mobility; SC, self‐care; UA, usual activities; PD, pain/discomfort; AD, anxiety/depression

The mean EQ VAS score for MSA was 59.5 (SD: 17.81) and the mean EQ‐5D‐5L index value was .558 (SD: .276). The EQ‐5D‐5L index values and EQ VAS scores of participants in the different subgroups are displayed in Table 2. Female patients and patients with frontal lobe dysfunction, cognitive impairment, depression, anxiety, excessive daytime sleepiness, PD‐SP, and fatigue had lower EQ‐5D‐5L index values. Female patients and patients with orthostatic hypotension, depression, anxiety, PD‐SP, and fatigue had lower EQ VAS scores. No significant differences in the EQ‐5D‐5L index values and EQ VAS scores were observed between patients with MSA‐C and MSA‐P. The frequencies of the five levels in the five dimensions of the EQ‐5D‐5L in all MSA patients and the two subtypes of patients are shown in Figure 1. A total of 92.1% of MSA patients reported problems with mobility, followed by a different number of patients having problems with usual activities (88.7%), self‐care (81.3%), anxiety/depression (72.1%), and pain/discomfort (53.9%). The frequencies of reported problems of each dimension in MSA‐C and MSA‐P are displayed in Figure 2. The most common problem was problems with mobility in both MSA‐C and MSA‐P patients, followed by problems with usual activities, self‐care, anxiety/depression, and pain/discomfort. The frequency of reported problems with pain/discomfort in patients with MSA‐P was significantly higher than that in patients with MSA‐C, while the frequency of reported problems with mobility in patients with MSA‐P was significantly lower than that in patients with MSA‐C.

**TABLE 2:** EQ‐5D‐5L index values and EQ VAS scores regarding the clinical features in patients with MSA

	Groups	Number	EQ‐5D‐5L index value	*p*‐value	EQ VAS score	*p*‐value
Sex	Male	212	.687 (.485−.792)	<.001*	60 (50−70)	.014*
	Female	168	.513 (.281−.734)		60 (50−70)	
Subtypes	MSA‐C	205	.606 (.363−.760)	.498	60 (50−70)	.148
	MSA‐P	175	.638 (.363−.792)		60 (50−70)	
OH	No	225	.638 (.400−.783)	.118	60 (50−70)	.045*
	Yes	155	.571 (.347−.778)		60 (50−70)	
FLD	No	164	.691 (.446−.792)	<.001*	60 (50−70)	.051
	Yes	216	.566 (.290−.734)		60 (50−70)	
CI	No	213	.691 (.444−.792)	<.001*	60 (50−70)	.112
	Yes	167	.513 (.208−.724)		60 (50−70)	
Depressive mood	No	130	.749 (.595−.841)	<.001*	70 (58.75−80)	<.001*
	Yes	250	.511 (.259−.723)		60 (50−70)	
Anxiety mood	No	109	.748 (.592−.841)	<.001*	70 (60−80)	<.001*
	Yes	271	.513 (.296−.734)		60 (50−70)	
EDS	No	300	.638 (.375−.792)	.021*	60 (50−70)	.327
	Yes	80	.513 (.245−.734)		60 (50−70)	
RBD	No	171	.642 (.438−.783)	.089	60 (50−70)	.318
	Yes	209	.513 (.281−.734)		60 (50−70)	
PD‐SP	No	304	.669 (.441−.792)	<.001*	60 (50−70)	<.001*
	Yes	76	.431 (.134−.573)		60 (50−70)	
Fatigue	No	152	.734 (.521−.841)	<.001*	60 (50−80)	<.001*
	Yes	228	.513 (.301−.724)		60 (50−70)	

*Note*: EQ‐5D‐5L index values and EQ VAS scores are displayed as the median and interquartile range. Higher EQ‐5D‐5L index values and EQ VAS scores indicate a better health‐related quality of life. Mann–Whitney test was used to compare the EQ‐5D‐5L index values and EQ VAS scores between two levels of each subgroup.

Abbreviations: CI, cognitive impairment; EDS, Excessive daytime sleepiness; EQ‐5D‐5L, the five‐level EuroQol five‐dimension questionnaire; EQ VAS, visual analog scale; FLD, frontal lobe dysfunction; MSA, multiple system atrophy; MSA‐C, MSA with predominately cerebellar ataxia; MSA‐P, MSA with predominately parkinsonism; OH, orthostatic hypotension; PD‐SP, Parkinson's disease‐related sleep problems; RBD, Rapid eye movement sleep behavioral disorder.

^*^Significant at level .05.

### 3.3 Factors related to EQ‐5D‐5L index values and EQ VAS scores in all MSA patients and patients with the two subtypes

Spearman's analysis showed a moderate correlation between the EQ VAS scores and total UMSARS, the Fatigue Severity Scale, the Parkinson's Disease Sleep Scale 2nd version, the Hamilton Depression Rating Scale., and the Hamilton Anxiety Rating Scale scores in all MSA patients. The total UMSARS score was strongly correlated with the EQ‐5D‐5L index values. Scores for the frontal assessment battery, MoCA, the Fatigue Severity Scale, the Parkinson's Disease Sleep Scale 2nd version, the Hamilton Depression Rating Scale, and the Hamilton Anxiety Rating Scale had weak to moderate correlations with EQ‐5D‐5L index values for all MSA patients (Table S2).

Mixture regression models and linear regression models showed the determinants of EQ‐5D‐5L index values and EQ VAS scores in total MSA and two subtypes of patients. In the total MSA patients, fatigue, PD‐SP, depressive mood, anxiety mood, and greater total UMSARS score were determinants for lower EQ‐5D‐5L index values. In addition, fatigue, depressive mood, and greater total UMSARS score were determinants for the lower EQ VAS scores. Total UMSARS score and PD‐SP were associated with lower EQ‐5D‐5L index values in both MSA‐C and MSA‐P. Total UMSARS score and fatigue were determinants of lower EQ VAS scores in both MSA‐C and MSA‐P. However, some differences existed in the determinants for EQ‐5D‐5L index values and EQ VAS scores between patients with MSA‐C and MSA‐P. For example, fatigue was the determinant for lower EQ‐5D‐5L index values in patients with MSA‐P but not in patients with MSA‐C. More details are displayed in Tables 3 and 4.

**TABLE 3:** Mixture regression analysis of EQ‐5D‐5L index values for total MSA group and the two subtypes

	MSA					MSA‐C					MSA‐P				
Independent variables	Coef	*dy*/*dx*	SE	*p*	95% CI	Coef	*dy*/*dx*	SE	*p*	95% CI	Coef	*dy*/*dx*	SE	*p*	95% CI
UMSARS total	−.0464	−.0127	.0028	<.001*	[−.0518 to −.0409]	−.0579	−.0146	.0052	<.001^*^	[−.0682 to −.0476]	−.0461	−.0127	.0039	<.001^*^	[−.0537 to −.0386]
OH	−.0227	−.0091	.0586	.6980	[−.1375 to .0921]	.1285	.0323	.0999	.1980	[−.0672 to .3242]	−.0227	−.0098	.0934	.8080	[−.2057 to .1603]
FLD	−.0886	−.0240	.0673	.1880	[−.2206 to .0433]	−.1109	−.0373	.1175	.3450	[−.3411 to .1193]	−.1544	−.0422	.1028	.1330	[−.3558 to .047]
CI	−.022	−.0034	.0682	.7470	[−.1557 to .1117]	−.0458	.0070	.1250	.7140	[−.2908 to .1992]	.0433	.0049	.0998	.6640	[−.1522 to .2389]
Fatigue	−.1735	−.0479	.0622	.0050*	[−.2954 to −.0516]	.0258	.0007	.1015	.7990	[−.1731 to .2247]	−.1931	−.0463	.0959	.0440*	[−.3811 to −.0051]
PD‐SP	−.2255	−.0675	.0745	.0020*	[−.3715 to −.0794]	−.5123	−.1361	.1394	<.001^*^	[−.7856 to −.239]	−.2335	−.0687	.1027	.0230*	[−.4347 to −.0323]
EDS	.0066	−.0039	.0702	.9250	[−.131 to .1443]	−.2147	−.0581	.1236	.0820	[−.457 to .0276]	.0292	.0001	.0998	.7700	[−.1666 to .2249]
RBD	.0237	.0090	.0605	.6950	[−.0948 to .1423]	.1109	.0313	.1086	.3070	[−.1019 to .3237]	−.0202	−.0062	.0864	.8150	[−.1896 to .1492]
Depressive mood	−.2015	−.0619	.0774	.0090*	[−.3531 to −.0498]	−.2567	−.0659	.1295	.0480^*^	[−.5105 to −.0028]	−.1916	−.0590	.1168	.1010	[−.4206 to .0373]
Anxiety mood	−.1746	−.0475	.0763	.0220*	[−.3242 to −.0251]	−.1644	−.0402	.1294	.2040	[−.4181 to .0893]	−.2758	−.0790	.1130	.0150*	[−.4973 to −.0543]
Model Wald *χ* ^2^	623.17			<.001*		323.37			<.001^*^		329.13			<.001*	

Abbreviation: CI, cognitive impairment; 95% CI, 95% confidence interval; EDS, Excessive daytime sleepiness; EQ‐5D‐5L, the five‐level EuroQol five‐dimension questionnaire; FLD, frontal lobe dysfunction; MSA, multiple system atrophy; MSA‐C, MSA with predominately cerebellar ataxia; MSA‐P, MSA with predominately parkinsonism; OH, orthostatic hypotension; PD‐SP, Parkinson's disease‐related sleep problems; RBD, rapid eye movement sleep behavioral disorder; SE, standard error; UMSARS, the Unified Multiple System Atrophy Rating Scale.

* Significant at level .05.

**TABLE 4:** Multivariate linear regression analysis of EQ VAS scores for total MSA group and the two subtypes

	MSA			MSA‐C			MSA‐P		
	*β*	SE	*p*‐value	*β*	SE	*p*‐value	*β*	SE	*p*‐value
UMSARS total	−0.410	0.069	<.001*	−0.426	0.097	<.001*	−0.466	0.093	<.001*
Fatigue	−4.794	1.762	.007*	−5.096	2.453	.039*	−5.057	2.438	.040*
Depressive mood	−4.483	1.852	.016*						
Anxiety mood							−5.967	2.522	.019*
Male				7.969	2.386	.001*			
Education	0.519	0.226	.022*						
F value	20.894		<.001*	13.264		<.001*	17.866		<.001*

Abbreviations: EQ VAS, visual analog scale; MSA, multiple system atrophy; MSA‐C, MSA with predominately cerebellar ataxia; MSA‐P, MSA with predominately parkinsonism; SE, standard error; UMSARS, the Unified Multiple System Atrophy Rating Scale.

*Significant at level .05.

## 4 DISCUSSION

Our research measured the HRQoL of patients with MSA using the EQ‐5D‐5L. We found that the mean EQ‐5D‐5L index value and EQ VAS score were .558 and 59.5, respectively. Problem with mobility was the most commonly reported problem, and the problem with pain/discomfort was the least common problem, which was consistent with the previous study using the EQ‐5D‐3L (Winter et al., 2011). In addition to disease severity, fatigue, PD‐SP, depressive mood, and anxious mood were found to be determinants of the EQ‐5D‐5L index values of MSA, and fatigue and depressive symptoms were determinants of the EQ VAS scores of MSA. Additionally, we found some differences in the determinants for HRQoL of patients with MSA between those with MSA‐C and those with MSA‐P.

The mean EQ‐5D‐5L index value (.558) and the mean EQ VAS score (59.5) of patients with MSA were lower than those of the Chinese healthy population (.943, 82.9) and those of similarly aged patients with coronary heart disease (.930, 71.13) and colorectal cancer (.617, no result for EQ VAS score) but were higher than those of Chinese patients with atrial fibrillation (.53, no result for EQ VAS score) and hemophilia (.51, 48.05) (Huang et al., 2018; Mei et al., 2021; Niu et al., 2022; Wang et al., 2018; Yang, Busschbach, Liu, & Luo, 2018). The mean index value (.558) and the mean EQ VAS score (59.5) in the current study were higher than the results of the European study using EQ‐5D‐3L (0.3 and 44.5, .29 and 53.3) (Higginson et al., 2012; Schrag et al., 2006). This may be explained by the different tools and disease duration among studies. There was a natural upward shift in index values for the EQ‐5D‐5L used in the current study, compared to that for the EQ‐5D‐3L used in the previous studies (Thompson & Turner, 2020). In addition, patients in our study had shorter disease duration than those in the previous studies (2.4 vs. 3.5−5.9 years) (Du et al., 2018; Higginson et al., 2012; Meissner et al., 2012; Schrag et al., 2006; Winter et al., 2011). The result of the current study provided important information for healthcare decision‐making on patients with early‐stage MSA. Meissner et al. found that patients with MSA had significantly decreased HRQoL after 11.5 months of follow‐up. According to the abovementioned evidence, we recommended that follow of the HRQoL of patients with MSA at the 1‐year or shorter interval to capture the change of HRQoL.

We found that there were some differences between patients with MSA‐P and MSA‐C. A higher frequency of problems with mobility was reported in patients with MSA‐C than in patients with MSA‐P in the current study. However, no difference in the mean scores of motor examination and global disability scale of UMSARS between the two subtypes was found. Our finding suggested that UMSARS has a limited ability to reflect mobility in patients with MSA‐C. In addition, we found that patients with MSA‐P reported more problems with pain/discomfort than patients with MSA‐C. This may be due to the more severe damage in the basal ganglia in MSA‐P than in MSA‐C (Lin, Xu, Hou, Yang, & Shang, 2020).

Depression was reported to be a determinant of the QoL of patients with MSA (Du et al., 2018; Torny et al., 2009; Winter et al., 2011; Zhang et al., 2019). In our study, depressive emotion was related to lower EQ‐5D‐5L index values and EQ VAS scores of patients with MSA. We also found that anxiety played a role in the decreased EQ‐5D‐5L index values in MSA and lower EQ VAS scores of MSA‐P (Du et al., 2018). Depression and anxiety were associated with a lower EQ‐5D‐5L index value and EQ VAS score in the Chinese elderly (Liao et al., 2021). A previous study found that more severe depressive symptoms were determinants of lower EQ VAS scores in MSA and progressive supranuclear palsy (Winter et al., 2011). In addition, a higher baseline anxiety level was a predictor of lower EQ‐5D‐3L index values and EQ VAS scores at the 3‐month and 1‐year follow‐ups in women who terminated their pregnancies (Toffol et al., 2016). Our previous study found that depression and anxiety were significantly related to longer disease duration and more severe disease severity in MSA, which may account for the association between mood symptoms and the EQ‐5D‐5L index value (Zhang et al., 2018). In addition, depression and anxiety were correlated with low self‐efficacy (Simonetti et al., 2021; Sympa et al., 2018), which may contribute to a low self‐rated EQ VAS score (Peters, Potter, Kelly, & Fitzpatrick, 2019). Our findings emphasized that the management of depressive and anxiety symptoms in MSA should be considered.

In the current study, we found that fatigue was negatively correlated with EQ‐5D‐5L index values and EQ VAS scores of MSA patients, which was consistent with our previous study evaluating the QoL of MSA patients using the PDQ‐39 (Zhang et al., 2017). A total of 29%–82% of MSA patients suffered from fatigue, which was significantly higher than that of patients with PD and healthy controls (Meyer et al., 2021). Fatigue negatively influenced the HRQoL but was only slightly captured by the EQ‐5D (Spronk, Polinder, Bonsel, Janssen, & Haagsma, 2021). A bolt‐on item addressing fatigue improved the informativity and decreased the ceiling effect of EQ‐5D‐5L (Spronk, Polinder, Bonsel, Janssen, & Haagsma, 2022). In addition, a bolt‐on item increased the explanatory power of EQ‐5D‐5L in the population with a high prevalence of fatigue and can capture the low HRQoL of patients with severe fatigue but slight disability (Spronk et al., 2022; Yang et al., 2015). So, EQ‐5D‐5L with a fatigue item may be preferred in patients with a high incidence of fatigue. The pathological mechanisms of fatigue are still uncertain. A study found that fatigue in MSA was related to a decrease in the 5‐hydroxytryptamine receptor in the raphe nuclei and brain stem (Meyer et al., 2021).

In our previous study, sleep‐related disorders, including PD‐SP, RBD, and excessive daytime sleepiness, were found to be associated with higher disease severity in patients with MSA (Lin et al., 2020). In the current study, PD‐SP but not RBD or excessive daytime sleepiness was found to be a determinant of the EQ‐5D‐5L index values in MSA patients. Since PD‐SP includes motor symptoms at night, PD symptoms at night, and disturbed sleep, the etiology of PD‐SP in MSA is complex and is still under research. Management of PD‐SP in MSA is necessary. Patients with OH had an increased risk of falls than those without OH and suffered from orthostatic symptoms such as syncope and shoulder pain upon standing (Fanciulli, Leys, Falup‐Pecurariu, Thijs, & Wenning, 2020). The severity of OH was positively related to the severity of the disease (Pavy‐Le Traon et al., 2016). These may result in lower HRQoL in patients with OH compared to those without OH in the current study.

This research had several limitations. First, the current study was a cross‐sectional research. We planned to follow up patients included in the current study at 1‐year interval to explore the longitudinal change of HRQoL in patients with MSA (Meissner et al., 2012). Second, the diagnosis of MSA was based on the clinical features but not the autopsy. Further study should build a confirmed autopsy cohort to study the HRQoL of MSA patients. Third, EQ‐5D‐5L was a general HRQoL measurement. Studies using the MSA HRQoL scale, a 40‐item scale developed for evaluating HRQoL in MSA, may provide a better breakdown of HRQoL. And we have started to use this scale in our evaluation.

## 5 CONCLUSION

The current research evaluated HRQoL in patients with MSA with the EQ‐5D‐5L. The determinants of lower EQ‐5D‐5L index values were greater total UMSARS, fatigue, PD‐SP, depressive mood, and anxiety symptoms scores, while those of lower EQ VAS scores were greater total UMSARS, fatigue, and depressive symptoms scores. In addition, depression was a determinant of HRQoL in MSA‐C but not in MSA‐P, while anxiety was related to HRQoL in MSA‐P but not in MSA‐C. This research provided important information on the HRQoL and potential determinants of MSA patients, helping guide the development of intervention strategies to improve their health status.

## AUTHOR CONTRIBUTIONS

Yi Xiao contributed in organization and execution of research project, study design, statistical analysis, writing of the draft, and review and critique of the manuscript. Lingyu Zhang contributed in organization and execution of research project, study design, statistical analysis, and review and critique of the manuscript. Qianqian Wei contributed in organization and execution of research project and design of the statistical analysis. Tianmi Yang, Kuncheng Liu, Ruwei Ou, Yanbing Hou, and Junyu Lin helped in organization and execution of research project. Huifang Shang helped in conception, organization, and execution of research project and review and critique of the manuscript.

## CONFLICT OF INTEREST

The authors declare no conflict of interest.

### PEER REVIEW

The peer review history for this article is available at: https://publons.com/publon/10.1002/brb3.2774.

## Supporting information

Supplementary Table 1 Data distribution of EQ‐5D‐5L index value in Total MSA and two subtypes
Supplementray Table 2 Spearman correlation between EQ VAS score, EQ‐5D‐5L index value and clinical characteristics of patients with MSA.

## Data Availability

The data used in the current study are available from the corresponding author on reasonable request.
