# Supplementary material for: Health‐related quality of life in patients with multiple system atrophy using the EQ‐5D‐5L
Source: Brain Behav. 2022 Sep 19;12(10):e2774. doi: 10.1002/brb3.2774 (PMC9575615; doi:10.1002/brb3.2774)
Supplement: Supplementary file 1 — Supplementary Table 1 Data distribution of EQ‐5D‐5L index value in Total MSA and two subtypes Supplementray Table 2 Spearman correlation between EQ VAS score, EQ‐5D‐5L index value and clinical characteristics of patients with MSA. [file BRB3-12-e2774-s001.docx]

Supplementary Table 1 Data distribution of EQ-5D-5L index value in Total MSA and two subtypes

|  | Mean | SD | Min | Max | Skewness | SD | Kurtosis | SD |
| --- | --- | --- | --- | --- | --- | --- | --- | --- |
| Total MSA | 0.558 | 0.276 | -0.391 | 1.000 | -0.648 | 0.125 | -0.324 | 0.250 |
| MSA-P | 0.553 | 0.266 | -0.391 | 1.000 | -0.649 | 0.170 | -0.351 | 0.338 |
| MSA-C | 0.564 | 0.289 | -0.227 | 1.000 | -0.658 | 0.184 | -0.304 | 0.365 |

Abbreviations: EQ-5D-5L: the five-level EuroQol five-dimensions questionnaire. MSA: multiple system atrophy. MSA-P: MSA with predominately parkinsonism. MSA-C: MSA with predominately cerebellar ataxia. SD: standard deviation.

Supplementray Table 2 Spearman correlation between EQ VAS score, EQ-5D-5L index value and clinical characteristics of patients with MSA.

|  | EQ VAS score (r) | P value | EQ-5D-5L  index value (r) | P value |
| --- | --- | --- | --- | --- |
| UMSARS-total | -0.379 | <0.001* | -0.717 | <0.001* |
| FAB | 0.144 | 0.005* | 0.246 | <0.001* |
| MoCA | 0.154 | 0.003* | 0.289 | <0.001* |
| FSS | -0.253 | <0.001* | -0.380 | <0.001* |
| PDSS-2 | -0.304 | <0.001* | -0.356 | <0.001* |
| ESS | -0.029 | 0.579 | -0.101 | 0.049* |
| RBDSQ | -0.075 | 0.142 | -0.110 | 0.031* |
| HAMD | -0.380 | <0.001* | -0.564 | <0.001* |
| HAMA | -0.359 | <0.001* | -0.479 | <0.001* |

The correlation coefficient (r) ≥ 0.8 is defined as a very strong correlation; 0.60–0.79, a strong correlation; 0.4–0.59, a moderate correlation; 0.20–0.39, a weak correlation; and r ≤ 0.19, a negligible correlation. *Significant at level 0.05.

Abbreviations: MSA: multiple system atrophy. EQ-5D-5L: the five-level EuroQol five-dimension questionnaire. EQ VAS: visual analog scale. UMSARS: the Unified Multiple System Atrophy Rating Scale. FAB: the frontal assessment battery. MoCA: the Montreal Cognitive Assessment. FSS: the Fatigue Severity Scale. PDSS-2: the Parkinson's Disease Sleep Scale 2nd version. ESS: the Epworth Sleepiness Scale. RBDSQ: Rapid Eye Movement Sleep Behavior Disorder Screening Questionnaire. HAMD: the Hamilton Depression Rating Scale. HAMA: the Hamilton Anxiety Rating Scale.
